# Testing marine regional-scale hypotheses along the Yucatan continental shelf using soft-bottom macrofauna

**DOI:** 10.7717/peerj.8227

**Published:** 2020-01-02

**Authors:** Ivan Hernandez-Avila, Frank A. Ocaña, Daniel Pech

**Affiliations:** 1 Laboratorio de Biodiversidad Marina y Cambio Climático (BIOMARCCA), El Colegio de la Frontera Sur, Campeche, Mexico; 2 Facultad de Ciencias Naturales, Universidad Autónoma del Carmen, Ciudad del Carmen, Campeche, Mexico

**Keywords:** Biodiversity, Gulf of Mexico, Continental Shelf, Macrofauna, Functional traits, Species assemblage, Calcareous sediments, Western Caribbean

## Abstract

Different hypotheses related to the regional-scale configuration of the Yucatan Continental Shelf (YCS) between the Gulf of Mexico (GoM) and the Caribbean Sea have been proposed. Hypotheses regarding its regional boundaries include: (i) an ecoregional boundary at Catoche Cape, dividing the Western Caribbean and the Southern GoM ecoregions; and (ii) a boundary within the Southern GoM ecoregion at 89°W, separating the West and Mid-Yucatan areas. We tested the hypothesis of no variation in benthic macrofaunal assemblages between regions delimited by the former boundaries using the species and functional traits of soft-bottom macrofauna. We considered that the depth and temporal environmental dynamics might interact with regional variations, generating complex benthic community patterns. The data were collected over five years (2010–2012, 2015–2016) at 86 stations (*N* = 1, 017 samples, 10–270 m depth), comprising 1,327 species with 45 combinations of functional traits. The variation in species composition and functional trait assemblages were both consistent with the occurrence of three separate regions in the Yucatan Peninsula (West Yucatan, Mid-Yucatan and Western Caribbean). This regional configuration was consistent with changes in assemblage structure and depth zonation as well as temporal variation. Along with spatial and temporal variation, diversity diminished with depth and different regions exhibited contrasting patterns in this regard. Our results suggest that the spatial and temporal variation of soft-bottom macrofauna at YCS demonstrate the complex organization of a carbonate shelf encompassing different regions, which may represent transitional regions between the Caribbean and the GoM.

## Introduction

Ecoregions are defined as areas with relatively homogeneous communities that are distinct from adjacent systems (Spalding et al., 2007) and that are also affected by environmental conditions, such as currents, upwellings, primary productivity and sediment, among others. The delimitation of ecoregions enables us to understand biogeographical processes, large-scale diversity patterns and their environmental drivers (Paganelli, Marchini & Occhipinti-Ambrogi, 2012; Williams et al., 2015) and to define conservation priorities (Selig et al., 2014; Tear et al., 2014; Beger et al., 2015).

A comprehensive definition of the configuration of marine ecoregions has been proposed by Spalding et al. (2007) based on biogeographical data and environmental conditions. Since then, new evidence has emerged to either support or challenge this means of accounting for changes in the composition of assemblages (Williams et al., 2015) and even benthic deep-water assemblages (Hernández-Ávila, 2014; Hernández-Ávila et al., 2018) as well as genetic connectivity (DeBoer et al., 2014). Moreover, one current debate focuses on the delimitation of some ecoregional boundaries (Carpenter et al., 2011). For instance, a distinct regional configuration has been proposed for the Gulf of Mexico based on chlorophyll-a concentration patterns (Salmerón-García et al., 2011), which may help to redefine ecoregions in smaller areas. Nevertheless, the effects of depth on spatial variation at a regional scale remain poorly explored. There is a broad consensus that the environmental drivers that influence the distribution of benthos change with depth, leading to a gradient effect on the dynamics of the abundance and distribution of benthic assemblages from shallow to deep water (Zajac, 2008; McArthur et al., 2010). Testing different regional variation hypotheses and including depth in spatial analyses of marine benthos may assist in the identification of ecological variation at regional scales.

Soft-bottom marine assemblages are useful models for testing meso- and large-scale variation hypotheses because they can respond to changes in the main environmental factors used to define marine biogeographical areas, such as oceanographic conditions, depth, organic carbon inputs and sedimentary provinces (Snelgrove & Butman, 1994). Ecosystem functioning is associated with the biodiversity of soft-bottom habitats (Snelgrove et al., 2014; Strong et al., 2015), which can be studied according to the diversity of species and variations in their functional biological traits (Menezes, Baird & Soares, 2010). Analyzing biological traits may prove helpful in detecting ecosystem patterns and functioning and anthropogenic disturbances (Bremner, 2008; Suding et al., 2008; Vinagre et al., 2017) as well as the response of an assemblage to environmental conditions (Menezes, Baird & Soares, 2010; Paganelli, Marchini & Occhipinti-Ambrogi, 2012; Van der Linden et al., 2012).

According to Spalding et al. (2007), the Yucatan Shelf separates two marine ecoregions: the Southern Gulf of Mexico (GoM) and the Western Caribbean. The boundary between these two regions follows the northeastern contour of the Yucatan Shelf from Catoche Cape (21.6°N 87.1°W), which is bounded in the north by the Greater Antilles ecoregion. The Southern GoM ecoregion extends up to the Tamaulipas coast, where it meets the Northern GoM ecoregion. However, Salmerón-García et al. (2011) have proposed a method of regionalizing the GoM based on the regional pattern of chlorophyll-*a*, which suggests that both the Northern and Southern GoM ecoregions can be subdivided into smaller regions. The regional setting of Salmerón-García et al. (2011) includes the separation of coastal Yucatan areas (<approximately 50 m deep) between the Tabasco-Campeche shelves and a section of the Campeche Bank extending up to approximately 89°W.

Although different regional settings of the Yucatan area have been accepted in biogeographical analyses of marine assemblages (Francisco-Ramos & Arias-González, 2013; Williams et al., 2015), a degree of uncertainty exists regarding the marine communities along the Yucatan Peninsula. The aim of this paper is to test the hypothesis of no variation in macrofaunal assemblages between regions at the Yucatan Continental Shelf (YCS) through analyzing their patterns in terms of species composition and functional traits. The combined analysis of species and functional assemblages may offer a more integrated view of large-scale variations in marine communities by identifying different variation patterns between ecoregions with both depth and time. Given that the extent of coastal influence decreases with depth (Weissberger et al., 2008; Zalmon et al., 2013), assemblage structures may differ among ecoregions due to bottom topography. The samples for statistical analysis comprise multiannual data (2010, 2011, 2012, 2015 and 2016) collected along the depth range of the YCS that consider both temporal variations and the effects of depth on the composition of assemblages.

## Materials and Methods

### Study area and regional hypotheses

The YCS is a carbonate shelf extending over 100–300 km from the shoreline, covering an area of approximately 57,000 km^2^ and composed of sedimentary deposits of medium- to fine-grained carbonate sand (Balsam & Beeson, 2003; Logan et al., 1969). Owing to the karstic nature of the Yucatan Peninsula, most of the freshwater input (8. 6 ×10^6^ m^3^km^−1^yr^−1^) in the area comes from groundwater (Hanshaw & Back, 1980), affecting nutrient input and phytoplankton communities (Slomp & Van Cappellen, 2004; Troccoli-Ghinaglia et al., 2010). The Yucatan Shelf hosts various coral reefs near the coast and at the shelf margin (Zarco-Perello et al., 2013) as well as coastal lagoons and mangroves (Hernández-Guevara, Pech & Ardisson, 2008) along the shoreline. The major currents in the area include the GoM loop current, which primarily affects the eastern and northern sections of the Yucatan Peninsula, and westerly currents along the west coast (Zavala-Hidalgo, Morey & O’Brien, 2003). Historically, the YCS has been subjected to hurricanes (Boose et al., 2003).

**Figure 1:**
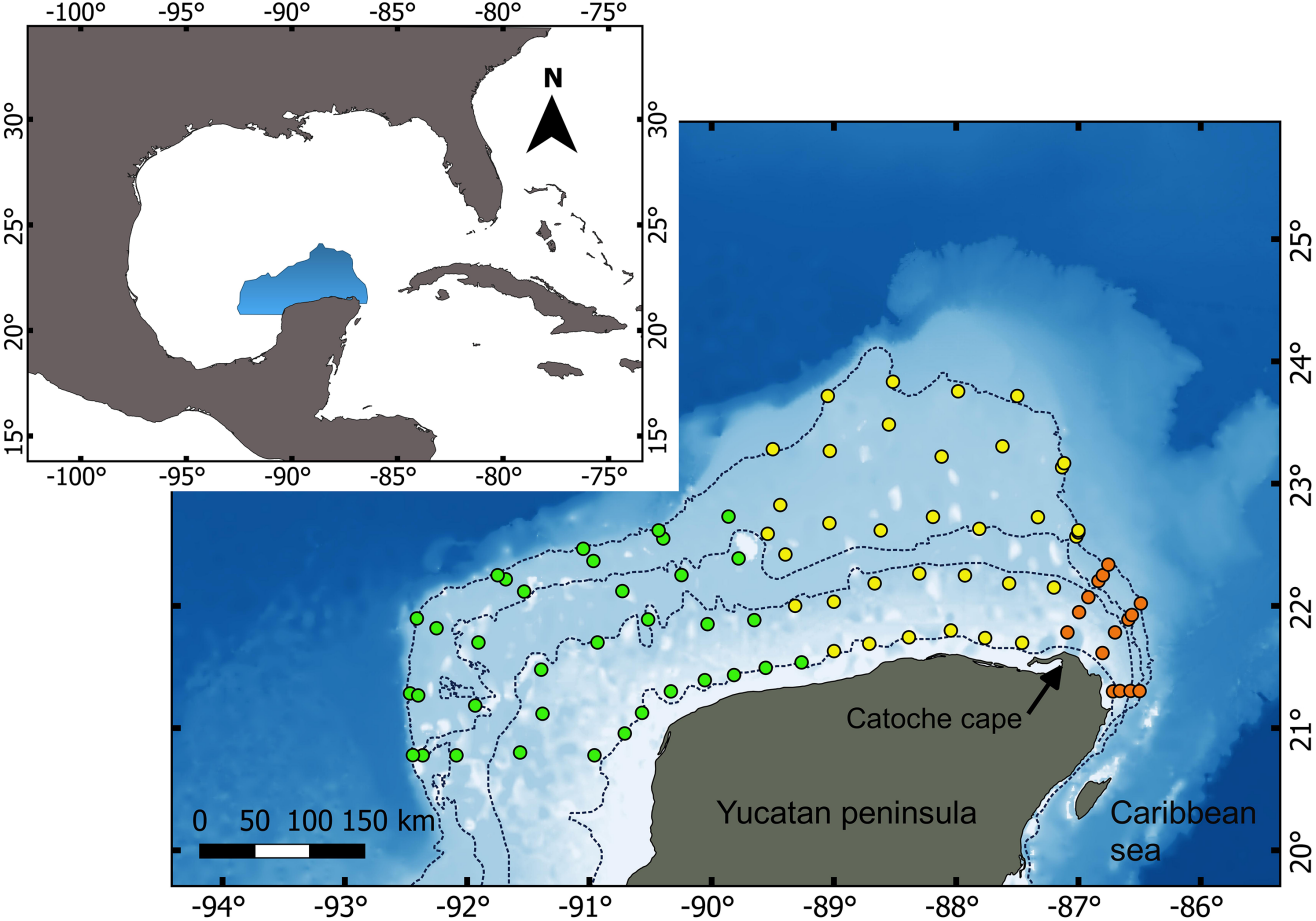
Study area and sampled stations included on five oceanographic cruises (2010, 2011, 2012, 2015, 2016) for macrofauna collections. Colors of stations are according regional-scale hypotheses tested in this study. Green circles, West-Yucatan; yellow circles, Mid-Yucatan; orange circles, Western Caribbean. Broken lines represent general isobaths for 10, 50, 100 and 200 m.

The regional-scale differences tested in this study include the division proposed by Spalding et al. (2007) between the Western Caribbean and the Southern GoM (Fig. 1). The northwestern boundary of the Southern GoM ecoregion is located at 23°N 97.8°W and is characterized by lower variation in the sea surface temperature during the winter than that observed in the Northern GoM ecoregion (Spalding et al., 2007; Wilkinson et al., 2009). The Southern GoM ecoregion also includes a subdivision between the YCS and Tabasco Shelf at around 92°W, in the Campeche Canyon (Lara-Lara et al., 2008; Wilkinson et al., 2009). According to this configuration, the western section of the YCS from Catoche Cape is considered as a single regional unit within the Southern GoM ecoregion (Yáñez Arancibia & Day, 2004; Zavala-Hidalgo & Fernandez-Eguiarte, 2007; Lara-Lara et al., 2008; Wilkinson et al., 2009).

An additional regional division of the Southern GoM, based on spatial variations in chlorophyll-*a* concentration, has been proposed by Salmerón-García et al. (2011) and includes a boundary at around 89°W, thereby separating a region in the centre of the YCS from the western section that includes Campeche Bank and the Tabasco Shelf. However, this regional setting does not include the boundary at Catoche Cape proposed by Spalding et al. (2007). Moreover, Salmerón-García et al. (2011) have proposed another ecoregion in the outer section of the YCS, defined by processes associated with the transition between coastal and oceanic zones (Ruíz-Castillo et al., 2016).

We compared macrofaunal assemblages across three regions proposed in previous works: (a) West Yucatan, as part of the Southern GoM region proposed by Spalding et al. (2007), including locations west of 89°W according to Salmerón-García et al. (2011); (b) Mid-Yucatan, located from the eastern boundary of the West Yucatan region to the Catoche Cape boundary, suggested by Spalding et al. (2007); and (c) the Western Caribbean, located east of Catoche Cape. By comparing regional differences in soft-bottom macrofaunal assemblages, we tested the hypothesis of a lack of regional-scale variation in benthic macrofaunal assemblages on the Yucatan Peninsula according to the boundaries proposed by Spalding et al. (2007), Salmerón-García et al. (2011) or both.

### Sample collection

Soft-bottom sediments were sampled as part of an integrated oceanographic survey on the YCS, from 2010 to 2012 and 2015 to 2016, to determine the biological baseline of the shelf. Sampling was conducted during five GoMEX oceanographic expeditions (GoMEX 1, 11 to 21 September, 2010; GoMEX 2, 23 September to 3 October, 2011; GoMEX 3, 27 November to 8 December, 2012; GoMEX 4, 2 to 20 November, 2015; and GoMEX 5, 25 August to 8 September, 2016) onboard the B/O Justo Sierra. The sampling approach for the benthic macrofauna consisted of sediment samples collected from 83 sampling points distributed in 16 transects along the depth gradient (Fig. 1).

All samples were collected using a 0.25-m^2^ Smith Mcintyre sediment grab, collecting three 10 cm random cores per grab at each station. Benthic macrofauna were recovered from the first 15 cm of the sediments using a 500 µm sieve. Once recovered, the organisms were pre-fixed using 15% magnesium chloride solution for 15 min and then fixed in a 4% formaldehyde buffer solution. Benthic macrofauna were identified to the lowest taxonomic level possible, typically to species or morphospecies level. All procedures from data sampling to database analysis, including taxonomic determination, were conducted following a quality assurance/quality control (QA/QC) protocol (D. Pech, 2018, unpublished data) developed by the Biodiversidad Marina y Cambio Climático (BIOMARCCA) laboratory.

**Table 1:** Traits and categories used to describe functional groups of soft-bottom macrofauna from the Yucatan continental shelf. Modified from Beauchard et al. (2017). Categories codes in brackets are shown in Fig. 2, Table 5 and Table S2.

Trait	Category	Response features	Effect features
Structural fragility	Soft and flexible (So)	Sensibility to physical damage	Generation of biogenic carbonate
	Hard shell protection (Sh)		
	Rigid exoskeleton (Ex)		
Motility	Mobile (Mo)	Adult dispersal, foraging mode, ability to escape predation	Habitat modification, bioturbation
	Limited motility (Lo)		
	Sessile (Se)		
Living position	Infaunal (In)	Sediment structure and epi/infaunal colonization	Habitat modification, bioturbation
	Epifaunal (Ep)		
Reproduction/ Development mode	Direct development with limited dispersion (Dd)	Juvenile survival and recruitment success	
	Parental care of eggs or planktonic larvae (Ec)		
	Indirect development, no parental care or planktonic larvae (Id)		
Feeding mode	Filter/suspension (Fe)	Food availability, primary productivity, carbon transport	Demographic control (predation), nutrient cycling
	Deposit (De)		
	Herbivore (He)		
	Predator (Pr)		
	Scavenger (Sc)		
	Omnivore (Om)		

### Identification of functional traits

Five functional traits commonly used for benthic fauna (Costello et al., 2015) were selected to represent the ecological functioning or the response of taxa to their environment. These traits were divided into categories (Table 1) and used to generate a binary taxa × trait matrix, where one (1) represented the fit of the taxa to the dominant trait category and zero (0) represented “no” fit (Beauchard et al., 2017). Traits were mostly identified based on a literature review conducted for each species or close relative taxa (genera or within family levels). Some traits were estimated based on direct observations (i.e., the presence and type of skeleton or feeding structures) and by consulting experts. One category per trait was selected to identify species trait combinations, with the exception of feeding trait, which presented one or two categories (e.g., predator and scavenger). Species with records of more than two feeding categories were included as omnivorous. The combination of trait categories was used to construct a trait combination × sample matrix by merging the abundance of species fitted in the same combination of functional traits for each sample.

### Data treatment

### Estimation of taxa and functional trait contributions to the assemblage

To combine the contributions of the taxa to the general community composition based on their presence, abundance and richness, we calculated a taxon contribution index (TCI) as follows:

(1) \begin{eqnarray*}TC{I}_{i}= \frac{p{r}_{i}+a{r}_{i}+r{r}_{i}}{3} (1)\end{eqnarray*}

where pr_i_, ar_i_, and rr_i_ were the corresponding presence, abundance and richness ratios of “i” taxa, respectively, estimated as:

(2) \begin{eqnarray*}& & p{r}_{i}= \frac{ \left( \frac{\sum presenc{e}_{i}}{\sum samples} \right) }{\sum p{r}_{i}} \end{eqnarray*}

(3) \begin{eqnarray*}& & a{r}_{i}= \frac{{n}_{i}}{N} \end{eqnarray*}

(4) \begin{eqnarray*}& & r{r}_{i}= \frac{\sum specie{s}_{i}}{\sum species} \end{eqnarray*}

The TCI_i_ values ranged from 0 to 1. The maximum score was obtained when taxon “i” was always present in the sampling data set and contained all possible species and individuals. Taxa with higher species and levels of abundance could exhibit high TCI scores, but this was also true of taxa with few species where their occurrence in the sampling set was high. The TCI was not estimated in the place of traditional indices, but rather as a general combined reference for the taxon’s relevance within the community composition. The index was estimated using Class-level taxonomic references, with the exception of crustaceans, which we included at the Order level. Similarly, to estimate the relative relevance of trait combinations, the functional trait index (FTI) was estimated based on the ratio of species richness and the presence and abundance of each combination of functional traits. The estimations were based on Eqs. (1)–(4), substituting the term “i” with the functional trait combination.

### Estimation of species diversity

To compare species richness between depth ranges, accumulation species curves (obtained by sampling and the Chao 1 estimator) were plotted as functions of depth ranges using ESTIMATES (Colwell, 2013). Estimations were conducted per depth interval (<50 m; 50–100 m; >100 m), extrapolated to 400 samples (for the species cumulative curve) and based on 100 permutations. Extrapolations and confidence intervals were estimated using the derivation of the Bernoulli product model (Chao et al., 2009; Clowell et al., 2012). Estimations were conducted using data obtained from all cruises. Differences in species richness as a function of depth were tested by conducting t-tests based on the parameters obtained by ESTIMATES. In addition, we compared the confidence intervals of each curve (Colwell, Mao & Chang, 2004; Clowell et al., 2012).

### Testing regional-scale variations in species composition and functional traits

Two similarity matrices were calculated using the Bray-Curtis Index based on both the abundance of each species and the abundance of each combination of functional traits. The null hypothesis of non-differences in the assemblage compositions between regions, depth zones and years was tested by conducting permutational multivariate analysis of variance (PERMANOVA) (Anderson, 2001) for both cases. The PERMANOVA tests included Region and Year as factors, with sampling depth (10–270 m) as the covariable. Region (Western Caribbean, Mid-Yucatan, West Yucatan) and Year (2010, 2011, 2012, 2015, 2016) were included as fixed factors. In addition, sampling station was included as a nested factor (16–41 stations per Region). Three pseudoreplicates were considered for each sampling point. The null model was tested using 9,999 permutations of residuals under the reduced model. For fixed factors, PERMANOVA t-tests were used to conduct pairwise comparisons. The effect of depth on assemblage structure was also explored using canonical analysis of principal coordinates (CAP), using station centroids against mean depth. The significance of the first-square canonical correlations was tested using 999 permutations (Clarke & Gorley, 2015).

The interactions between temporal variation (Year) and regional variation (Region) in terms of assemblage composition were explored using second-stage analyses (Clarke et al., 2006) for both species and functional trait assemblages. Regarding the absence of an interaction effect between Year and Region, high second-stage correlations between regional matrices of temporal variation were expected (Clarke et al., 2006; Clarke & Gorley, 2015). The distribution trends of species assemblages and functional trait assemblages were represented by the bootstrap average of centroids in multidimensional scaling plots (MDS) using 95% confidence intervals and 30 bootstraps per group (Clarke & Gorley, 2015).

### Identifying influential species

The original species assemblage was reduced to a subset of species that could represent the overall pattern using a BVStep routine (Clarke & Warwick, 1998; Clarke & Gorley, 2015). Similarity data matrices with different combinations of species subsets were compared with the similarity matrix containing all taxa to identify the smallest group of species that could best describe most of the pattern in the full data set. An iterative species exclusion-inclusion process was performed during the BVStep routine to obtain the minimum number of species from the species subset that could represent a similarity matrix that was highly correlated with the original similarity matrix. The Spearman’s rank coefficient was used to compare similarity matrices using a threshold of *ρ* > 0.90 as the minimum level of correlation between all taxa and species subset matrices. BVStep analyses were performed several times (over 30 trials) to ensure that the best species subset was selected.

## Results

### General taxonomic and functional composition

**Figure 2:**
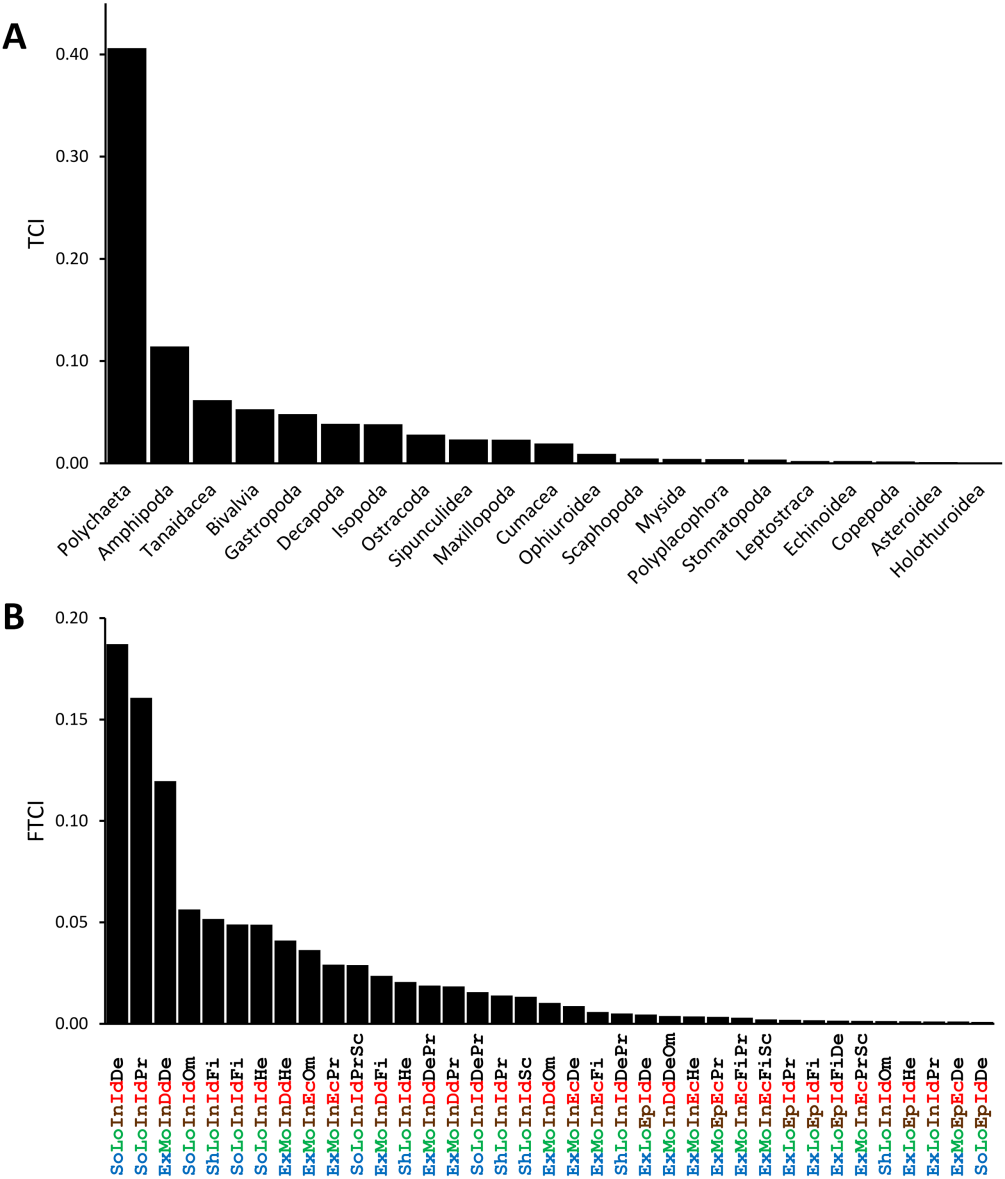
Taxonomic (A) and functional (B) contribution indexes estimated on the five year sampling collection (2010–2012, 2015–2016) of the Yucatan shelf (10–270 m depth). Colors in trait codification refers to structural fragility (blue), motility (green), living position (brow), reproduction/development mode (red) and feeding (black). Reference codes for trait categories in Table 1.

The benthic macrofauna collected from the YCS comprised 19,892 individuals across 1,329 species. The major taxa included Annelida (Polychaeta), Arthropoda (Crustacea), Mollusca, Echinodermata, Nemertea and Sipuncula. Polychaeta exhibited the highest abundance and species diversity (Fig. 2A), followed by crustaceans (particularly Amphipoda, Tanaidacea, Isopoda, Decapoda, Ostracoda and Cumacea) and Mollusca (bivalves and gastropods). Polychaeta, Crustacea and Mollusca accounted for 97% of species and 79% of individuals.

The combination of trait categories produced 45 functional trait combinations for the YCS soft-bottom macrofauna. Five functional trait combinations accounted for most of the specimens in terms of species and occurrence (Fig. 2B): (i) organisms with a soft, flexible body and limited motility, a deposit feeding mode, providing no parental care for their eggs and producing planktonic larvae; (ii) organisms similar to the first group, but with a predator feeding mode; (iii) organisms with a fragile exoskeleton and relatively high motility, a deposit feeding mode and direct development, with limited dispersion of their propagules; (iv) organisms with soft, flexible bodies and limited motility, with planktonic larvae and omnivorous feeding; and (v) shelled organisms with limited motility, a filter feeding mode and planktonic larvae.

### Trends in species richness

**Figure 3:**
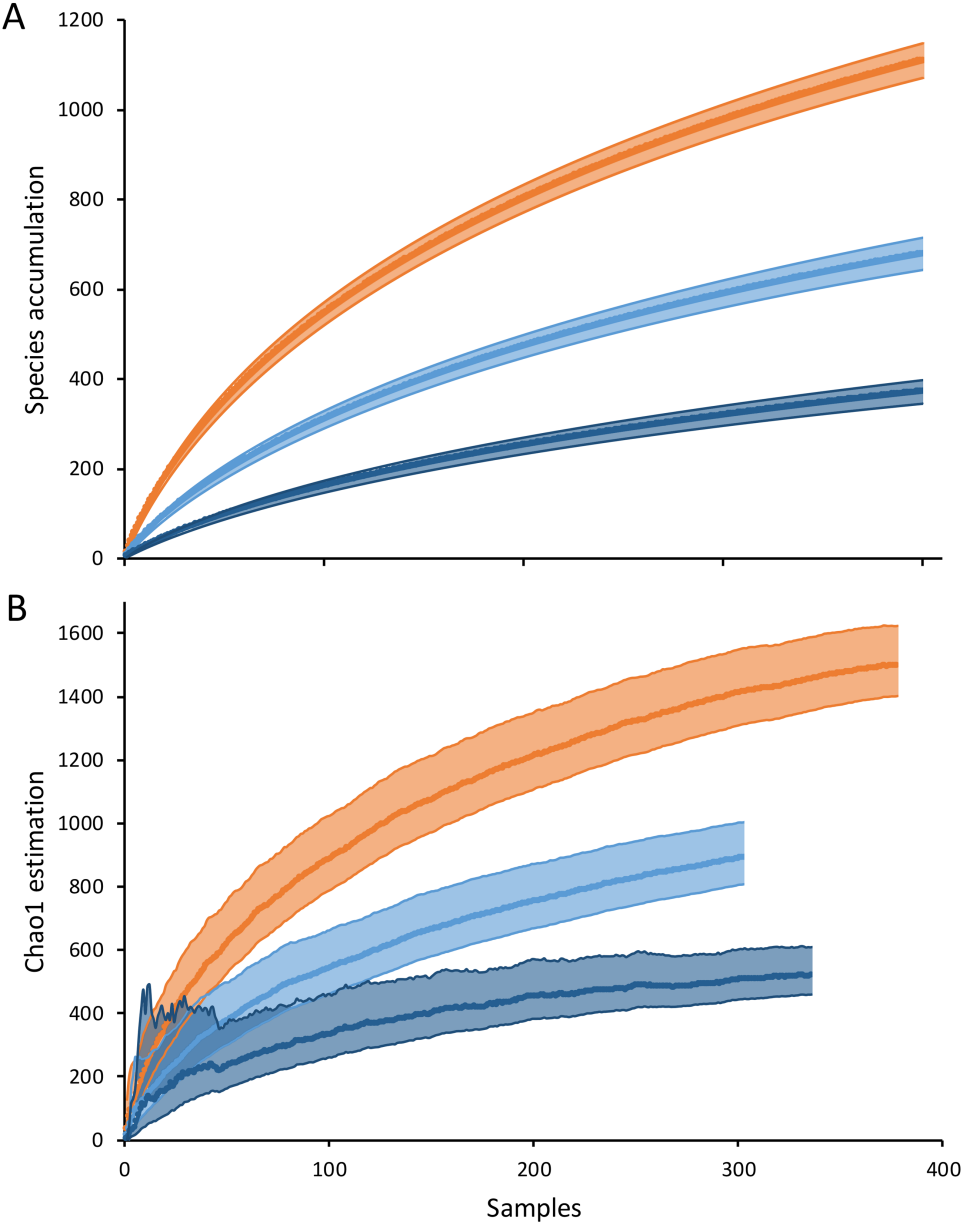
Cumulative curve of (A) species per sample (extrapolated to 380 samples) and (B) Chao1 estimator at different depth ranges of the Yucatan shelf. Red, 10–50 m depth; ligth blue, 50–100 depth; dark blue, >100 m depth. Shaded areas represent 95% confidence intervals for each estimation.

Overall, the cumulative species diversity (1,526.2 ± 24.5 spp., extrapolated to 1,500 samples) was slightly lower than the Chao 1 estimator (1,742.7 ± 52.5 spp.), with both exhibiting an almost asymptotic pattern. Higher species richness was estimated at shallower depths (<50 m, 1,109.5 ± 19.2 spp.) than that at 50–100 m (678.5 ± 18.2 spp.) and >100 m (371.8 ± 13.4 spp.). The Chao 1 estimator showed a similar trend with depth, but higher species richness scores (shallow depth: 1,497.5 ± 56.5 spp., *n* = 378; mid-depth: 891 ±49.7 spp., *n* = 303; and >100 m: 518.9 ± 37.9 spp., *n* = 336). Differences between depths were detected using both the cumulative species curve and the Chao 1 estimator (spp. accum: shallow *vs* 50-100 m *t*-test = 325.9, 50–100 m *vs* >100 m *t*-test = 279.3; Chao1: shallow *vs* 50–100 m *t*-test = 149.8, 50–100 m *vs* > 100 m *t*-test = 113.9; *p* < 0.001, all cases) (Fig. 3). The decrease in diversity with depth at the YCS was also consistent across all sampling years.

### Changes in species composition and functional traits along the Yucatan continental shelf

According to the PERMANOVA test, variations in assemblage structure based on species composition and functional traits exhibited consistent responses to spatial (region, depth) and temporal (year) factors and their combinations (Table 2). The significant correlation between station centroids and depth (species assemblage: Δ^2^ = 0.832; functional trait assemblage: Δ^2^ = 0.664; *p* = 0.001 both cases) suggest an effect of depth on species and functional assemblages. In both types of assemblage, the effect of depth differed by Region and Year, denoting varied zonation patterns. Significant interactions were detected between region and year. *A posteriori* analyses indicated significant changes in species composition between all years in the three ecoregions. Functional trait assemblages also presented variations in most of the pairwise analyses between years, with different patterns between regions (Table 3). The two-stage analyses of region per depth range centroids exhibited low correlations between years (Spearman’s correlation, species assemblage *ρ* < 0.428, functional trait assemblage *ρ* < 0.518), supporting the occurrence of different temporal variation patterns occurring at each region and depth range. The variation of the temporal trends between the assemblages of the regions constituted the interactive effects detected between Region and Year.

**Table 2:** PERMANOVA of species (A) and functional traits (B) assemblages (Bray–Curtis similarity) of the Yucatan Shelf. Sources of variation on interactions represented by their first letters. *p*-value < 0.05 in bold.

Source	df	SS	MS	Pseudo-F	P(perm)	√CV
	**A. Species assemblage**					
Depth	1	1.64E + 05	1.64E + 05	27.574	**0.0001**	12.477
Region	2	31,128	15,564	2.329	**0.0007**	5.3604
Year	4	85,057	21,264	6.6871	**0.0001**	9.5551
Station(R)	83	5.32E + 05	6,405.3	2.1464	**0.0001**	17.134
D x R	2	11,926	5,963.1	1.9982	**0.0001**	8.4093
D x Y	4	32,614	8,153.5	2.7322	**0.0001**	5.5769
R x Y	8	66,744	8,343	2.7957	**0.0001**	10.162
D x R x Y	8	42,821	5,352.6	1.7936	**0.0001**	7.084
Residual	904	2.70E + 06	2,984.3			54.628
Total	1,016	3.66E + 06				
	**B. Functional traits assemblage**					
Depth	1	2.21E + 05	2.21E + 05	44.756	**0.0001**	14.577
Region	2	29,459	14,730	2.6046	**0.0033**	5.409
Year	4	51,704	12,926	6.2535	**0.0001**	7.4041
Station(R)	83	4.48E + 05	5,401.7	2.8968	**0.0001**	17.421
D x R	2	11,465	5,732.5	3.0742	**0.0002**	9.5822
D x Y	4	31,728	7,931.9	4.2536	**0.0001**	6.0419
R x Y	8	42,473	5,309.1	2.8471	**0.0001**	8.1472
D x R x Y	8	30,975	3,871.9	2.0764	**0.0001**	6.5215
Residual	904	1.69E + 06	1,864.7			43.183
Total	1,016	2.55E + 06				

**Table 3:** *p*-values of PERNAMOVA pairwise test for change in assemblage composition, species and functional traits assemblages, between sampling years at each region. Sampling depth included as a covariable. *p*-values < 0.05 (Boferroni corrected) in bold. A, West Yucatan, B, Mid Yucatan, C, Western Caribbean.

Species assemblage					Functional trait assemblage				
	2010	2011	2012	2015			2010	2011	2012	2015
	**A. West-Yucatan**								
2010						2010				
2011	**<0.001**					2011	**0.0040**			
2012	**<0.001**	**<0.001**				2012	**<0.001**	**0.0010**		
2015	**<0.001**	**<0.001**	**<0.001**			2015	0.0557	**0.0243**	**0.0045**	
2016	**<0.001**	**<0.001**	**<0.001**	**<0.001**		2016	**<0.001**	**0.0015**	**0.0144**	**0.0015**
	**B. Mid-Yucatan**								
2010						2010				
2011	**<0.001**					2011	0.0831			
2012	**<0.001**	**<0.001**				2012	**0.0437**	**0.0095**		
2015	**<0.001**	**<0.001**	**<0.001**			2015	**0.0105**	0.0528	**<0.001**	
2016	**<0.001**	**<0.001**	**<0.001**	**<0.001**		2016	0.0993	0.1279	**0.0025**	**0.0010**
	**C. Western Caribbean**								
2010						2010				
2011	**<0.001**					2011	**<0.001**			
2012	**0.0049**	**0.0015**				2012	**0.0065**	0.2019		
2015	**<0.001**	**<0.001**	**<0.001**			2015	**<0.001**	0.2372	**<0.001**	
2016	**0.0114**	**0.011**	**<0.001**	**<0.001**		2016	**0.0045**	0.8256	**0.0257**	**<0.001**

**Figure 4:**
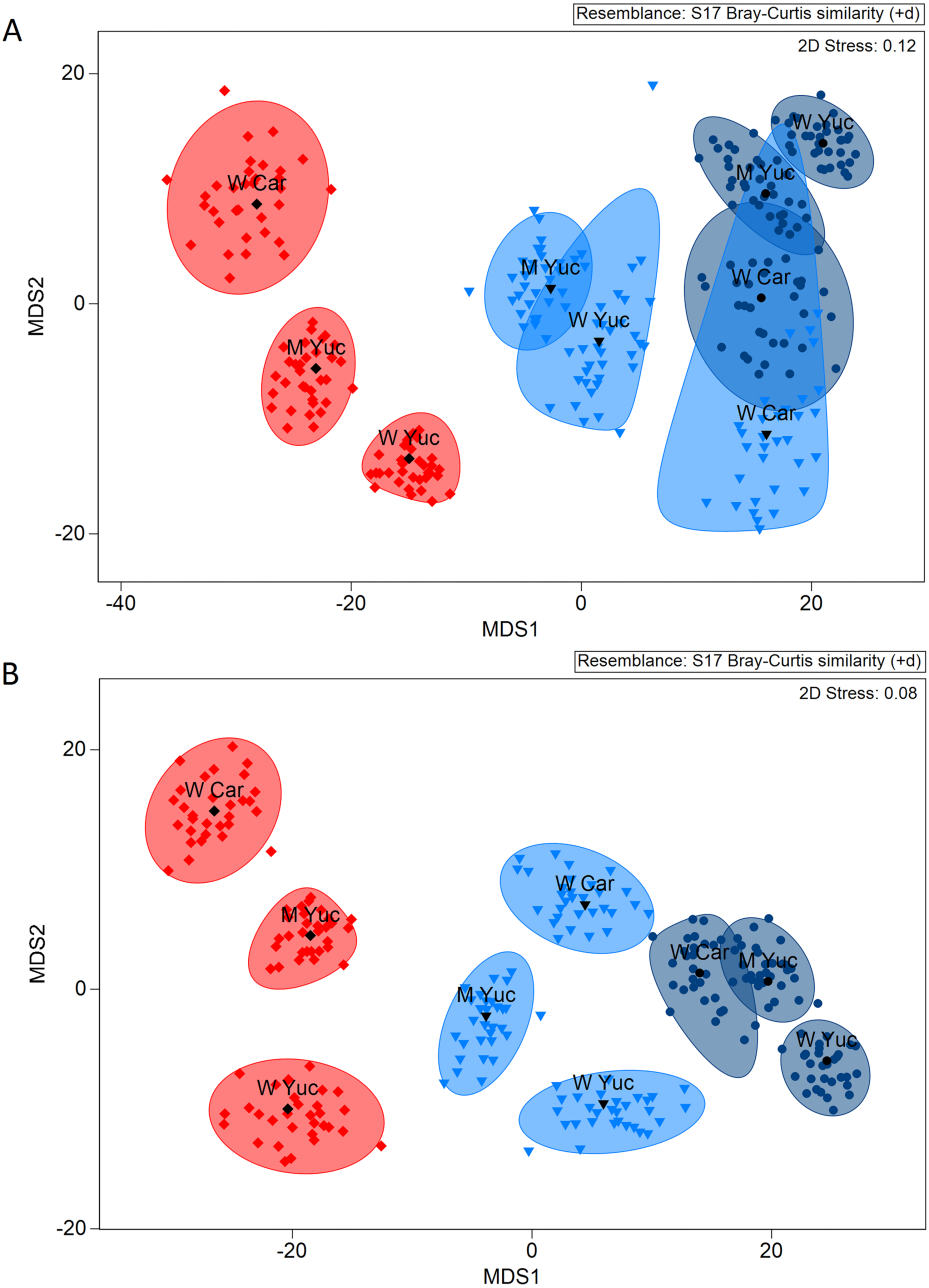
Bootstrapping centroids of assemblage composition in regions and depth ranges for species assemblages (A) and functional trait assemblages (B). Red, 10–50 m; light blue, 50–100 m; dark blue, >100 m depth. W Yuc, West Yucatan; M Yuc, Mid-Yucatan; W Car, Western Caribbean. Black symbols represent the centroid averages (33 bootstraps per group) for each combination. Shaded areas represent 95% confidence interval per group.

The *a posteriori* analysis performed for each depth range revealed that the species assemblage showed differences between regions at all depths (Table 4). For the functional trait assemblage, significant differences were found for the upper shelf, but the spatial variation decreased at lower sections. At shallow depths (<50 m), significant differences between the Western Caribbean and both Mid- and West Yucatan were found. At mid-depth (50-100 m), differences between Mid- and West Yucatan were detected, while no differences were found between these regions and the Western Caribbean. Beyond 100 m, no differences were detected between the three regions (Table 4). The MDS plot based on centroids indicated that the regional differences observed in the species and functional trait assemblages at the shallow and mid-depths diminished at depths exceeding 100 m (Fig. 4).

**Table 4:** PERMANOVA pairwise test for changes in assemblage composition (species and functional traits) between regions on the Yucatan Shelf at each depth range. *p*-values <0.05 (Bonferroni corrected) in bold. (A) 10–50 m depth. (B) 50–100 m depth. (C) >100 m depth.

Pairwise test	Species assemblage		Functional traits assemblage	
Depth range	t	p(perm)	t	p(perm)
	**A. 10–50 m**			
W-Yucatan vs M-Yucatan	1.7256	**0.0003**	1.4514	0.0805
W-Yucatan vs W-Caribbean	1.7025	**0.0003**	2.0673	**0.001**
M-Yucatan vs W-Caribbean	1.5515	**0.0003**	1.555	**0.0455**
	**B. 50–100 m**			
W-Yucatan vs M-Yucatan	1.459	**0.01**	1.8195	**0.0212**
W-Yucatan vs W-Caribbean	1.8483	**0.0003**	1.4654	0.1943
M-Yucatan vs W-Caribbean	1.6645	**0.0003**	1.3466	0.21290
	**C. >100 m**			
W-Yucatan vs M-Yucatan	1.2953	**0.0484**	0.744	0.9764
W-Yucatan vs W-Caribbean	1.6683	**0.0003**	1.2736	0.3893
M-Yucatan vs W-Caribbean	1.3402	**0.0285**	0.87652	0.9069

### Taxa associated with community structure and their variation

BVStep analysis allowed us to reduce the former 1,327 species into a subset of 60 species that reproduced the observed patterns (Spearman’s correlation *ρ* = 0.942). The removal of the species subset from the former matrix reduced the correlation with the general matrix (*ρ* = 0.72). These results indicated a lack of structural redundancy in the data set, with the new 60-taxa subset reproducing the spatial–temporal variation of species detected in the YCS.

The reduced group of representative taxa included polychaete spionids (*Prionospio cristata*, *Exogone dispar*, *Aonidella dayi*, *A. mayaguensis* and *Paraprionospio pinnata*), cirratulids (*Aphelochaeta* sp. and *Kirkegaardia dorsobranchialis*), ophelids (*Armandia agilis* and *A. maculata*), paraonids (*Aricidea simplex* and *A. suecica*), pilargids (*Litocorsa antennata*) and others; *amphipod* Aoridae (*Bemlops* sp. and *Pleosiolembos ovalipes*), Ampeliscidae (*Ampelisca vadorum*), Unciolidae (*Rudilemboides naglei*) and Phoxocephalidae (*Rhepoxynius* sp. and *Eobrolgus spinosus*); and tanaidaceans Leptocheliidae (*Alloleptochelia longimana* and *Chondrochelia dubia*), Apseudidae (*Apseudes* sp.) and Metapseudidae (*Calozodion wadei*). Single species of other major taxa in the representative subset included the ostracod Rutidermatidae *Rutiderma aff. cohenae*, the Isopoda Hyssuridae *Xenanthura brevitelson* and non-identified sipunculans (Table 5 and Table S1). Although the trend for most species was a reduction in abundance with depth, some species of the subset exhibited an increase in abundance from 50–100 m or at 100–270 m, such as Spionidae (*Aonidella dayi*, *Spiophanes bombyx* and *Prionospio dayi*) and Pilargidae (*Litocorsa antennata*).

### Variation in functional traits by ecoregion and depth range

Consistent with the PERMANOVA test, variations in the distribution of functional traits were observed. In general, reductions in the abundance of most trait combinations were observed from shallower to greater depths. The depth pattern differed between trait combinations and regions, as observed in Table 5 and Table S2. Significant variations in trait combinations between the Western Caribbean and both Mid-Yucatan and West Yucatan were detected at 10–50 m (Table 4), where the Western Caribbean was dominated by infaunal soft-bodied predators while West Yucatan was dominated by two types of infaunal deposit feeders (organisms with soft-bodies and limited motility with indirect development; and exoskeleton-bearing, motile organisms with direct development). In Mid-Yucatan, infauna were dominated by deposit feeders, predators and herbivores (including soft-bodied species with limited motility and indirect development, and motile, exoskeleton-bearing species that undergo direct development). At a depth of 50–100 m, the differences between Mid- and West Yucatan were associated with an increase in soft-bodied predators, herbivores and exoskeleton-bearing detritivores in the former.

**Table 5:** Abundance (ind 0.1m -2 , mean ± se) of 10 relevant taxa from the representative sub-set of 60 the taxa that reproduce the observed patterns, and the 10 most abundant combination of functional traits of the macrofauna at YCS. Abundance is estimated according to depth range and regions. Abbreviations in brackets, Pol, Polychaeta; Amp, Amphipoda; Tan, Tanaidacea; Cop, Copepoda; Gas, Gastropoda; Sip, Sipuncula. Colors in functional trait codification refers to structural fragility (blue), motility (green), living position (brown), reproduction/development mode (red) and feeding mode (black). Reference codes for functional traits are the same used in Table 1. Information on the subset of the 60 representative taxa are show in Table S1 and from the 20 most abundant combinations of functional traits in Table S2.

Depth range	10–50 m			50–100 m			>100 m		
Regions	West-Yucatan (*n* = 116)	Mid-Yucatan (*n* = 164)	Western Caribbean (*n* = 112)	West-Yucatan (*n* = 88)	Mid-Yucatan (*n* = 147)	Western Caribbean (*n* = 74)	West-Yucatan (*n* = 112)	Mid-Yucatan (*n* = 74)	Western Caribbean (*n* = 95)
Taxa									
*Prionospio cristata* (Pol: Spionidae)	11.8 ± 2.1	19.6 ± 5.6	19.2 ± 5.5	15.6 ± 3.7	3.6 ± 1.0	0.3 ± 0.3	0.3 ± 0.2	0.3 ± 0.2	0.2 ± 0.2
*Aphelochaeta* sp. 1 (Pol: Cirratulidae)	63.6 ± 35.5	0.4 ± 0.2	0.8 ± 0.4	0.5 ± 0.2	0.7 ± 0.4	1 ± 0.7	0 ± 0	0.3 ± 0.2	0.5 ± 0.3
*Armandia agilis* (Pol: Opheliidae)	15.6 ± 4.0	10.3 ± 3.0	3.7 ± 1.4	3.4 ± 1.1	1.6 ± 0.4	0.7 ± 0.5	0.4 ± 0.2	0.1 ± 0.1	0.9 ± 0.9
*Fabricia* sp. 1 (Pol: Fabriciidae)	26.2 ± 6.7	5.9 ± 1.4	4.9 ± 1.5	1.5 ± 0.5	0.8 ± 0.3	0.3 ± 0.3	0.2 ± 0.1	0.1 ± 0.1	1.6 ± 1.4
*Protodorvillea kefersteini* (Pol: Dorvilleidae)	0.4 ± 0.3	1.0 ± 0.5	11.4 ± 3.1	0.2 ± 0.1	0.7 ± 0.3	0.3 ± 0.3	0 ± 0	0.3 ± 0.2	0.2 ± 0.2
*Plesiolembos ovalipes* (Amp: Aoridae)	9.26 ± 5.7	1.9 ± 1.2	2.8 ± 2.0	0 ± 0	0 ± 0	0 ± 0	0 ± 0	0 ± 0	0 ± 0
*Chondrochelia dubia* (Tan: Leptocheliidae)	14.5 ± 4.8	2.2 ± 0.6	15.4 ± 3.7	1.8 ± 0.5	1.9 ± 0.6	2.3 ± 1.5	0.7 ± 0.51	0.1 ± 0.1	0.2 ± 0.2
Harpacticoida (Cop)	7.0 ± 2.0	14.2 ± 3.6	16.2 ± 4.4	2.0 ± 0.7	3.2 ± 1.0	0.7 ± 0.5	0.4 ± 0.4	1.4 ± 0.7	0.7 ± 0.4
*Caecum pulchellum* (Gas: Caecidae)	7.1 ± 4.5	6.0 ± 3.5	0.3 ± 0.2	0 ± 0	0 ± 0	0 ± 0	0 ± 0	0 ± 0	0 ± 0
Sipunculidae (Sip)	2.1 ± 0.5	10.3 ± 5.4	23.1 ± 18.4	2.6 ± 1.2	1.8 ± 0.5	0.7 ± 0.7	1.9 ± 0.4	4.0 ± 1.3	2.5 ± 0.9
Funtional traits									
SoLoInIdDe	163.4 ± 56.0	61.0 ± 8.4	92.4 ± 16.0	36.9 ± 6.0	31.8 ± 4.2	24.3 ± 3.1	10.8 ± 1.6	19.3 ± 2.5	20.4 ± 2.7
SoLoInIdPr	46.8 ± 8.1	40.5 ± 5.0	124.4 ± 19.1	8.8 ± 1.6	21.4 ± 2.9	23.1 ± 4.5	7.3 ± 1.0	10.5 ± 1.4	12.6 ± 1.8
ExMoInDdDe	98.9 ± 17.1	49.4 ± 8.5	67.5 ± 12.9	15.2 ± 2.8	18.5 ± 2.2	13.1 ± 3.1	2.1 ± 0.8	1.7 ± 0.5	3.2 ± 0.8
SoLoInIdOm	23.6 ± 5.0	9.1 ± 1.7	11.1 ± 2.1	8.8 ± 2.2	10.4 ± 2.1	5.9 ± 2.2	3.6 ± 0.7	2.6 ± 0.6	2.4 ± 0.6
ShLoInIdFi	9.3 ± 1.7	16.2 ± 4.2	10.5 ± 2.6	5.1 ± 1.5	4.0 ± 0.9	2.4 ± 0.8	0.6 ± 0.3	2.1 ± 0.8	1.2 ± 0.6
SoLoInIdFi	44.8 ± 10.2	15.1 ± 3.4	28.1 ± 7.9	4.1 ± 1.0	6.8 ± 1.2	2.4 ± 0.8	0.8 ± 0.3	2.0 ± 0.5	2.1 ± 1.0
SoLoInIdHe	14.5 ± 2.2	7.9 ± 1.4	5.7 ± 1.2	12.0 ± 3.0	19.1 ± 3.1	5.7 ± 1.1	2.0 ± 0.4	3.9 ± 0.8	5.1 ± 0.9
ExMoInDdHe	17.0 ± 4.7	34.4 ± 8.7	38.4 ± 13.7	1.5 ± 0.6	4.5 ± 2.7	4.8 ± 1.9	0.1 ± 0.1	0.2 ± 0.1	1.5 ± 0.5
ExMoInEcOm	4.4 ± 0.9	13.0 ± 3.1	22.9 ± 4.5	2.5 ± 1.0	2.8 ± 0.8	6.5 ± 2.1	0.4 ± 0.2	1.0 ± 0.6	1.9 ± 0.8
ExMoInEcPr	17.6 ± 4.4	19.5 ± 5.7	7.8 ± 1.7	0.6 ± 0.3	1.9 ± 0.7	2.2 ± 1.0	0.4 ± 0.2	0.3 ± 0.2	0.4 ± 0.2

**Table 6:** Overall diversity of macrofaunal soft-bottom communities at continental shelf for some locations. Number of individuals and sampled area were included as a proxy of sample size. Only large sample size (>10^5^ individuals) were included for comparative purpose. Estimations of sampled area for each study were estimated based on number of samples reported on each study and sampling gear area.

Location	Species	Individuals	Sampled area (m^2^)	Source
Campos Basin, N RJ Stade, Brazil 12–97 m	1,112	24,165	2	Zalmon et al. (2013)
Southwest Florida Shelf	1,121	na^a^	na	Phillips, David & Keith (1990)
Sao Sebastiao Coast, Brazil 8–45 m	392	23,456	12	Pires-Vanin, Arasaki & Muniz (2013)
Cretan shelf, Grece 40–90 m	547	18,858	9.9	Karakassis & Eleftheriou (1997)
Portugal Shelf 13–195 m	737	30,000	14.5	Martins, Quintino & Rodrigues (2013)
Monterey Bay, Ca, USA, 30–95 m	ca 1,000	ca 100,000	15.4	Oliver et al. (2011)
Monterey Bay, Ca, USA, 100–150 m	ca 800	ca 40.000	5	Oliver et al. (2011)
Santa Maria (R1), Ca, USA, 90–92 m^a^	336–419	32,390–69,182	7.1	Hyland et al. (1991)
Santa Maria (R2), Ca, USA, 145–161 m^a^	275–358	21,559-38,582	4.8	Hyland et al. (1991)
Jossingfjord, Norway 107–185 m	358	38,569	7.2	Gray et al. (1997)
Frigg, Norway 70 m	592	29,0401	75	Gray et al. (1997)
Bass Strait, Australia 11–51 m	803	60,258	10.4	Gray et al. (1997)
Lochs Linnhe & Eil, Scotland 9–111 m	323	13,014	12	Pearson (1970)
Firth of Lorne, Etive and Creran, Scotland 24–117	ca 300	ca 37,000	11	Gage (1972)
Deception Island, Southern Ocean 5-15 m	69	24,384	0.4	Angulo-Preckler et al. (2017)
Northern Sicily, Italy 40–80 m	116	47,427	86.4	Romano et al. (2016)
Yucatan continental shelf 10–270 m	**1,329 ± 19.69**	**19,892**	**7.9**	**Present study**

**Notes.**

a From a large scale survey.

b Ranges of three sectors.

na, data not available.

## Discussion

The overall species diversity at the YCS was much higher than that of other soft-bottom macrofaunal shelf communities (Table 6). Only three locations with a comparable scale of diversity (>1,000 species) were identified: the Campos Basin of Rio de Janeiro (Zalmon et al., 2013), the Monterey Bay Continental Shelf (Oliver et al., 2011) (both of which have upwelling systems, like the YCS) and the carbonate shelf at the northern limit of the GoM (SW Florida) (Phillips, David & Keith, 1990). No other locations with high carbon input from riverine or upwelling origins exhibited a similar level of diversity. In the case of the YCS, the occurrence of different types of habitats (such as coral reefs, seagrass beds, coastal lagoons and groundwater inputs) could contribute to species diversity through some degree of connectivity between habitats. Most species in the three different assemblages observed here (69%) undergo indirect development with larval dispersal. The Yucatan Current could enhance larval connectivity by facilitating the recruitment of many species.

The general structure of the soft-bottom macrofauna was dominated by polychaetes and peracarid crustaceans, followed by molluscans (Bivalvia and Gastropoda), decapod crustaceans and ostracods, similar to the general patterns of the benthic communities in continental shelf habitats, although the lack of species-level classification conducted in previous assessments near our study area (Hernández-Arana et al., 2003; Escobar-Briones & Jimenez-Guadarrama, 2010) precludes direct comparison. Similar patterns have been detected using family-level information in previous studies on the western section of the YCS, with common dominant taxa including polychaetes from the families Spionidae, Syllidae, Paraonidae, Aspidosiphonidae, Sabellidae and Lumbrineridae (Hernández-Arana et al., 2003).

Consistent functional trait combinations that significantly contributed to the assemblages include: (i) infaunal organisms with a soft-flexible body, limited motility, larval dispersion and diverse feeding modes, such as deposit feeders, scavengers and predators (mainly polychaetes); and (ii) infaunal, exoskeleton-bearing motile species that undergo direct development with the deposit feeding or scavenging trophic mode (mainly peracarid crustaceans). Furthermore, the number of functional trait combinations decreased with depth, similar to the species diversity patterns.

The results for both the species and the functional trait assemblages were consistent with the regionalization of soft-bottom macrofauna at YCS. Biogeographical boundaries are related with shared species and diversity, but recent studies have highlighted the relevance of using a trait-based approach to describe marine biogeography (Brun, Payne & Kiørboe, 2016). Here, we demonstrate that trait analysis is a useful surrogate for depicting regional-scale differences in benthic assemblages, particularly for shallow depths. Despite detecting regional variations in species assemblages at all depth ranges, macrofaunal assemblages based on functional traits only exhibited regional variations in the shallow section of the shelf (10–50 m). This may have been a result of a regional variation in species with the same combination of functional traits. By including more traits and categories, it may be possible to achieve a greater resolution of functional trait combinations, facilitating the detection of spatial and temporal variation with higher accuracy.

The regional configuration of the soft-bottom benthic community represented a combination of the previous regional boundaries proposed for the area. The boundary proposed at Catoche Cape by Spalding et al. (2007), Lara-Lara et al. (2008) and Wilkinson et al. (2009), separating the Western Caribbean ecoregion, was consistent with our analyses. However, our data also suggest that the western section of YCS from Catoche Cape is not a single regional unit. Variations between Mid- and West Yucatan were identified, supporting these regions’ separation according to Salmerón-García et al. (2011). This result suggests that considering the Southern GoM ecoregion as a single unit may underestimate the ecological variability of the GoM. Detecting regional-scale variations in marine assemblages associated with environmental drivers in the Southern GoM would contribute to redefining smaller ecoregions within the natural regional-scale variation of the GoM. In the current case of the YCS, each area has a particular range of temporal and spatial (depth) variability, supporting the separation of three distinct ecoregions.

The effect of depth on assemblage composition differed among regions and years. The different benthic assemblage compositions could be associated with the varied shelf configurations observed along the YCS. Indeed, there is a short shelf close to the continent and a stepped slope in the Western Caribbean section. However, a very wide shelf is also present that extends 200–300 km from the continent with an almost steady slope at the West and Mid-Yucatan regions. Environmental conditions interacting with the physical configuration of the shelf may drive the different temporal dynamics of benthic communities, explaining the changes in temporal patterns and variations with depth between ecoregions.

Comparing the composition of assemblages at each depth range, both species and functional trait assemblages exhibited variations in assemblage structure between shallow (<50 m depth), mid- (50-100 m) and >100 m depths. According to Spalding et al. (2007), the ecoregions proposed for marine areas apply to coastal and shelf environments up to a depth of 200 m. Nonetheless, current data suggest that deeper shelf regions (>50 m deep) may exhibit less regional-scale variation in functional trait assemblages than shallow waters, although variations in assemblage compositions in the area were detected between the three ecoregions when considering the total depth range of the shelf. Comprehensive studies on marine biogeography have focused on large, global-scale depth ranges (Watling et al., 2013), but conducting a more precise separation of depth intervals for shelf environments might improve ecoregional classification, as demonstrated here.

**Figure 5:**
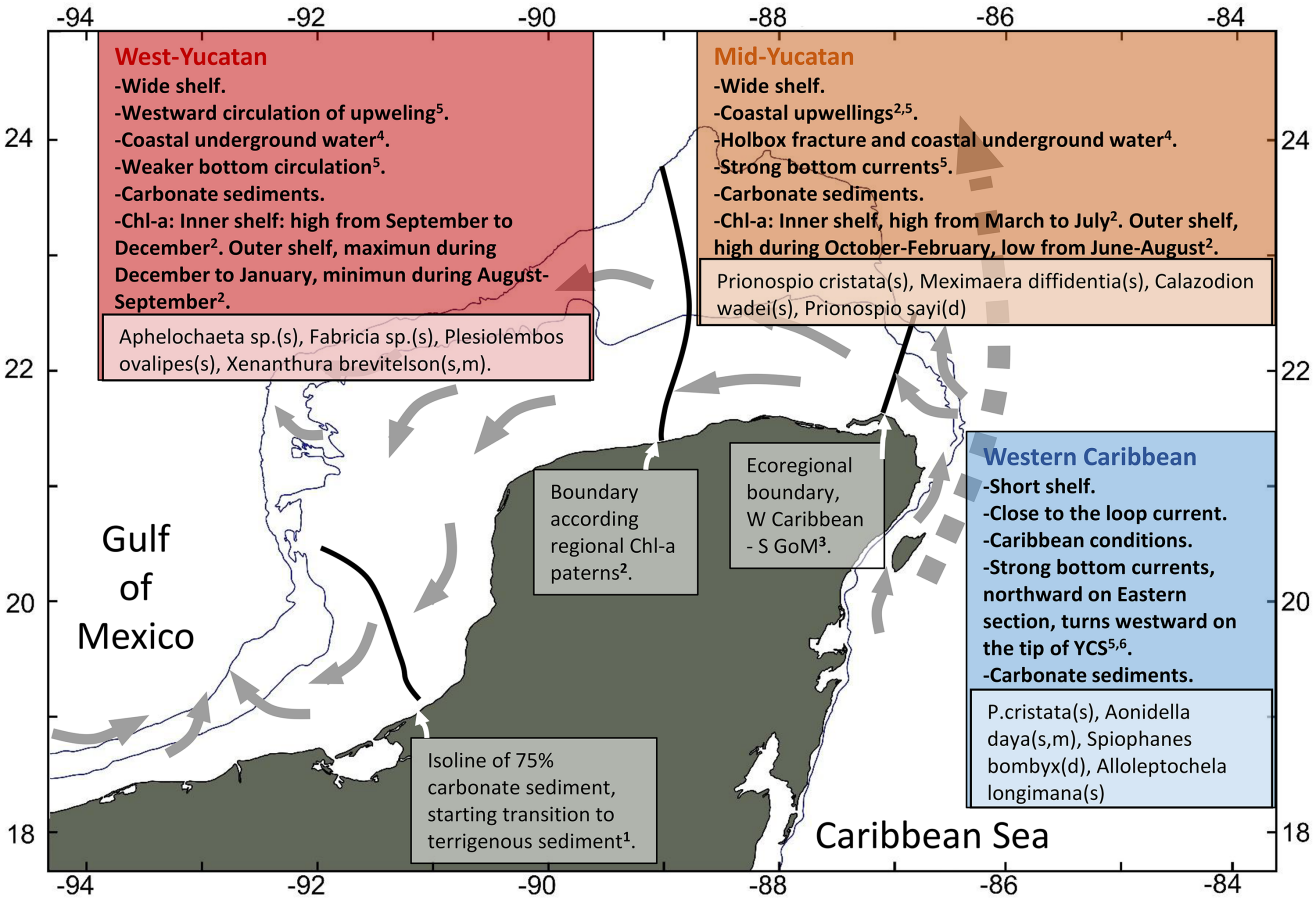
Conceptual model of tested regionalization at the YCS. The figure includes the 89°W boundary (Salmerón-García et al., 2011) extended to the outer shelf, and the ecoregional boundary between Western Caribbean and Southern GoM (Spalding et al., 2005). Also included is a suggested western limit base on the transition of carbonate sediments on the shelf and their effects on macroinfauna composition (Hernández-Arana et al., 2005). Gray arrows represent the loop current (dashed) and dominant currents on the shelf according to Kjerfve (1994), Zavala-Hidalgo, Morey & O’Brien (2003) and Lie-Yauw (2005). On inserted squares are species from the 60spp subset that were more abundant, comparing other regions. Brackets indicate depth range: s, 10–50 m; m, 50–100 m; d, >100 m. ^1^(Hernández-Arana et al., 2005), ^2^(Salmerón-García et al., 2011), ^3^(Spalding et al., 2007), ^4^(Pope, Ocampo & Duller, 1993), ^5^(Ruíz-Castillo et al., 2016), ^6^(Kjerfve, 1994).

The variations in assemblage composition may have been associated with differences in the water masses of the YCS. Water masses have already been suggested as the main drivers of the biogeographic structure of benthic shallow-water and continental-shelf species worldwide (Bellanger et al., 2012). The thermohaline conditions of the central coast of the Yucatan Peninsula may define major spatial oceanographic variations in areas where two types of bottom-water masses occur at shallow depths, including i) the local water mass (Yucatan Sea Water or YSW, temperature of 26-31 °C and salinity of 36.4–36.8), and ii) the upwelled Caribbean Subtropical Underwater (CSUW, <23 °C, salinity 36.25–36.75) (Enriquez et al., 2013). CSUW upwelling occurs at Cape Catoche and its effects extends offshore to the west up to 89.5°W, while the YSW occurs at both the central and western sections of the YCS, with a strong presence west of 89°W. Bottom water with low salinity was observed in the eastern section of the YCS due to the influence of submarine groundwater discharges (SGD) from the Holbox fractures occurring between 88 and 87.5°W (Pope, Ocampo & Duller, 1993). The combined effects of the CSUW upwelling and SGD may generate high primary productivity in the central section of the YCS, affecting the assemblages of soft-bottom communities, particularly those in the inner section of the shelf (Ruíz-Castillo et al., 2016). The westward circulation west of the 89°W upwelling may drive the extension of the upwelling towards the inner shelf, but with less influence on benthic communities. We suggest a conceptual model based on our results (Fig. 5) that indicates that soft-bottom communities in West Yucatan may be affected by YSW at shallow depths (0–50 m), Gulf Common Water (GCW) occurring from 0 to 250 m and by the wind-driven westward circulation of upwelled water (Ruíz-Castillo et al., 2016) and local SGD.

The non-differences between assemblages in the deeper section of the shelf (>100 m) may owe to the more homogeneous offshore oceanographic conditions. The lack of differences between deep water masses may generate more homogeneous bottom conditions than those observed at shallow depths. Moreover, local upwellings typically exert important inshore effects on bottom communities, but their extension to offshore waters depends on inshore-offshore transport (Ruíz-Castillo et al., 2016). The influence of inshore upwellings on deep offshore shelf communities can be lower than that on shallow-water communities. Beyond a depth of 100 m, SGD has little effect (if present) and bottom habitats in the area are affected by CSUW (150–250 m) and GCW (0–250 m) (Enriquez et al., 2013). According to Vázquez (2000) and Enriquez et al. (2013), GCW is the CSUW affected by mixing processes and local changes.

The delimitation of the study area did not allow us to determine the western limit of the West Yucatan region. However, owing to the scale of the spatial variations observed, its western limit could be located at the limit of the YCS, close to the Tabasco Shelf. In the western section of the Yucatan Continental Shelf (near Campeche Canyon, around 92°W), major differences in sedimentary settings have been observe, as they shift from a carbonate shelf to terrigenous sediments (Carranza-Edwards, Rosales-Hoz & Monreal-Gómez, 1993; Hernández-Arana et al., 2003; Hernández-Arana et al., 2005). These environmental conditions are also associated with shifts in the composition of soft-bottom communities (Hernández-Arana et al., 2003). This suggests that changes in the sedimentary provinces close to Campeche Canyon could generate additional biogeographical subdivisions in the western section of the YCS.

There is evidence to suggest that West Yucatan macrofauna change according to the dominant climatic periods occurring in the area (Hernández-Arana et al., 2003; Hernández-Guevara, Pech & Ardisson, 2008). Here, samples were collected during the late rainy seasons of 2010, 2011 and 2016 and the beginning of the cold front periods of 2012 and 2015. Some differences detected between the cruises were probably caused by differences between years and periods (between 2011–2012 and 2015–2016). Although seasonal variations in the coastal and shallow regions of the YCS are usually associated with continental run-off, wind stress and sea surface temperature (Hernández-Arana et al., 2003; Hernández-Guevara, Pech & Ardisson, 2008; Kuk-Dzul, Gold-Bouchot & Ardisson, 2012), climatic variations generate oceanographic conditions that can affect deeper waters. The oceanographic conditions of the Southern GoM during the rainy season include low wind stress (<5 m s^−1^), low mixed layer depth (<40 m) and low net primary production (<250 mg C m ^−2^day^−1^), while cold front conditions include high wind stress (>6 m s^−1^), a greater mixed layer depth (>80 m) and high primary productivity (>350 mgC m ^−2^day^−1^) (Müller-Karger et al., 2015). These contrasting scenarios may be associated with changes in carbon inputs to the bottom shelf, affecting the benthic infauna. Disentangling the potential seasonal variation from annual variation would allow for a better understanding of the dynamics of the benthic communities on the YCS.

In addition, annual variations were detected between the samples collected during the rainy season (2010 and 2011) as well as during the early cold front period (2012 and 2015). Although there is evidence that GoM shelf macrofauna change according to seasonal variations (Posey et al., 1996; Posey et al., 1998; Hernández-Arana et al., 2003), annual variations may also occur within the same period, but this aspect has been relatively less explored. In addition to seasonal variations, interannual climatological variations in the GoM are responsible for variations in phytoplankton biomass and primary production (Müller-Karger et al., 1991; Martínez-López & Zavala-Hidalgo, 2009; Müller-Karger et al., 2015). Variations in biomass generated at the sea surface would affect the input of carbon to the benthic shelf, influencing benthic communities. The potential seasonal and interannual variations associated with oceanographic conditions and phytoplankton biomass must be addressed by coupling environmental data and oceanographic models with the structure of species and functional trait assemblages.

## Conclusions

Our findings have demonstrated spatial, temporal and depth variations in the distribution of soft-bottom macrofaunal assemblages along the YCS. The spatial and temporal variations indicated the complex organization of carbonate shelf communities, which were previously believed to be relatively homogeneous environments, where major spatial and temporal changes can occur. The ecoregional boundaries of the Southern GoM (according to general environmental conditions) should be re-evaluated, considering the more precise delimitation of environmental and community assemblage variations. Our results suggest that the YCS contains different regions that appear to represent transitional regions between the Caribbean and the GoM. According to Olson & Dinerstein (1998), ecoregions are regional-scale conservation units because they encompass similar biological communities, and their boundaries approximately coincide with the area over which key ecological processes most strongly interact. Ecoregional delimitation may affect management actions at regional scales as well as the evaluation of their outcomes (Olson et al., 2001; Edgar et al., 2014). In the case of the large marine system of the GoM, a more precise definition of ecoregions based on the spatial and temporal complexity of the area is required. The inclusion of phytoplankton biomass and species assemblage patterns, in addition to soft-bottom macrofauna, may enhance biological and ecological arguments for defining ecoregional settings in the area. Testing the spatial variation of environmental conditions and biological assemblages along the Southern GoM ecoregion could engender an ecoregional configuration that is more consistent with the ecological variability of marine benthos in the area.

##  Supplemental Information

10.7717/peerj.8227/supp-1 Table S1 Abundance, Ind 0.1m ^2^ (mean ± se), of a representative sub-set of taxa (60 mostly at species level) of the macrofauna at YCS, Abundance is estimated according to depth ranges and regions.

10.7717/peerj.8227/supp-2 Table S2 Abundance, Ind 0.1m ^2^ (mean ± se), of 20 most relevant combinations functional traits of macrofauna at YCS, according regions and depth ranges Colors in trait codification refer to structural fragility (blue), motility (green), living position (brow), reproduction/development mode (red) and feeding mode (black). Reference codes for trait categories in Table 1.

10.7717/peerj.8227/supp-3 Supplemental Information 1 Raw data, functional trait assemblage per sample matrix Data points indicate collected abundance of specimens with the corresponding code of functional traits (first column, 46 files) at each sample (following 1,017 columns). Factors used for analyses on the bottom (files 49 to 52).

10.7717/peerj.8227/supp-4 Supplemental Information 2 Raw data, species per sample matrix Data points indicate collected abundance of specimens for each morphospecies (first column, 1,327 spp) at each sample (following 1,017 columns). Factors used for analyses on the bottom (files 1,330 to 1,333).

## Additional Information and Declarations

### Data Availability

The following information was supplied regarding data availability: The raw measurements are available in the Supplementary Files.
